# Structural, Surface, *in vitro* Bacterial Adhesion and Biofilm Formation Analysis of Three Dental Restorative Composites

**DOI:** 10.3390/ma8063221

**Published:** 2015-06-03

**Authors:** Maria T. Azam, Abdul S. Khan, Danish Muzzafar, Rani Faryal, Saadat A. Siddiqi, Riaz Ahmad, Aqif A. Chauhdry, Ihtesham U. Rehman

**Affiliations:** 1 Interdisciplinary Research Centre in Biomedical Materials, COMSATS Institute of Information Technology, Lahore 54000, Pakistan; E-Mails: mariaazam@ciitlahore.edu.pk (M.T.A.); saadatanwar@ciitlahore.edu.pk (S.A.S.); aqifanwar@ciitlahore.edu.pk (A.A.C.); 2 Faculty of Dentistry, SEGi University, Kota Damansara, Petaling Jaya 47810, Malaysia; E-Mail: danish9181@hotmail.com; 3 Department of Biosciences, COMSATS Institute of Information Technology, Islamabad 44000, Pakistan; E-Mail: ranifaryal@comsats.edu.pk; 4 The Centre for Advanced Studies in Physics, Government College University, Lahore 54000, Pakistan; E-Mail: ahriaz@gcu.edu.pk; 5 Department of Materials Science and Engineering, the Kroto Research Institute, the University of Sheffield, Sheffield S3 7HQ, UK; E-Mail: i.u.rehman@sheffield.ac.uk

**Keywords:** dental composites, spectroscopic analysis, bacterial adhesion, wettability, surface properties

## Abstract

This study was conducted to investigate the relationship between dental materials and bacterial adhesion on the grounds of their chemical composition and physical properties. Three commercially available dental restorative materials (Filtek™Z350, Filtek™P90 and Spectrum^®^TPH^®^) were structurally analyzed and their wettability and surface roughness were evaluated by using Fourier Transform Infrared Spectroscopy, Contact Angle Measurement and Atomic Force Microscopy, respectively. These materials were molded into discs and tested with three bacterial strains (*Staphylococcus aureus*, *Pseudomonas aeruginosa* and *Escherichia*) for microbial attachment. The bacterial adhesion was observed at different time intervals, *i.e.*, 0 h, 8 h, 24 h, 48 h and 72 h, along with Colony Forming Unit Count and Optical Density measurement of the media. It was found that all materials showed a degree of conversion with time intervals, *i.e.*, 0 h, 8 h, 24 h, 48 h and 72 h, which led to the availability of functional groups (N–H and C–H) that might promote adhesion. The trend in difference in the extent of bacterial adhesion can be related to particle size, chemical composition and surface wettability of the dental materials.

## 1. Introduction

Dentistry is a field that has progressed significantly in the last few decades. New techniques in restorative dentistry have changed conventional treatment methods [1]. However, dental restoration has been related to a high incidence of secondary caries [2,3,4,5]. For ideal application of restorative materials, parameters that define bacterial adhesion need to be researched prior to their clinical application [6]. An in-depth analysis of adhesion profiles of micro-flora inhabiting the oral cavity can prove to be productive in assessing the etiology of caries induction on dental restorative materials [7,8,9]. Bacterial adhesion on tooth and dental restorative material surfaces leads to dental caries and related periodontal diseases [10]. Secondary caries and infections associated with biofilm formation in the oral cavity on dental material surfaces [11] become augmented by gastritis and ulcers in certain cases. No correlation has been observed previously between adhesion of different bacterial strains on different types of composites [9,12]. However, several studies [13,14] reported a correlation between the roughness of dental materials and the accumulation of bacteria. For some time, several mono-species biofilm models have been available which accommodate typical oral microbes [15]. Streptococci have been frequently used as a caries model [16], where *in vivo* plaque microbiota is highly diverse and complex [17], and the oral fluid, too, is an essential component in the formation of dental biofilms [18].

Various dental composites have been recently introduced to the market including dimethacrylate based nano-composites and silorane based composites [19]. In this study, adhesion profiles of three bacterial (*Pseudomonas aerigunosa*, *Staphylococcus aureus and Eschereshia coli*) strains have been tested against three relatively newly developed commercial dental restorative materials. Two of these strains (*Pseudomonas aerigunosa* and *Staphylococcus aureus*) are linked to biofilm formation on dental materials and the natural tooth surface and they are associated with incidences of primary and secondary caries development [11,20,21]. *Escherichia coli* can be remotely involved in progression of dental diseases, especially in patients with chronic gastritis and acid reflux [22].

Materials that retard or inhibit bacterial attachment are deemed favorable for their use in dental restoration [23,24,25]. It is important that we take into consideration the commercially prepared dental composites and bacterial adhesion on their surfaces. Factors which may promote or inhibit adhesion such as chemical composition, surface roughness and the hydrophobic/hydrophilic nature of the restorative material were evaluated in this study. On the basis of surface chemistry, morphology of composite materials and bacterial strains, a general relationship could be found between the number of bacterial colonies showing adhesion and those showing mere attachment. It is expected that composite materials having greater surface roughness will help attached bacteria in retaining their position for a longer period of time. Similarly, particle size and degree of polymerization of composite material will also help to determine the status of bacterial adhesion. 

## 2. Results

### 2.1. Fourier Transform Infrared Spectroscopy (FTIR)

**Figure 1:**
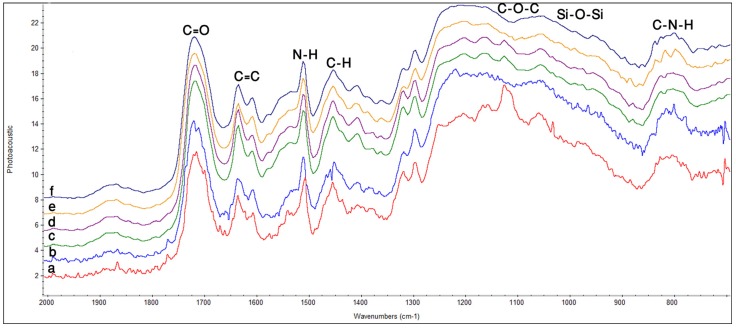
FTIR spectra of Z350 showing peaks for different functional groups at different time intervals; **(a**) before curing; (**b**) after curing 0 h; (**c**) 8 h; (**d**) 24 h; (**e**) 48 h; (**f**) 72 h.

**Z350:** In Figure 1a, spectra collected before curing displayed a peak at 1711 cm^−1^ that can be assigned to free carbonyl (C=O) stretch in resin polymer. The peak at 1452 cm^−1^ attributed to scissoring vibration of C–H presented in all the constituent monomers. Similarly, peaks at 1390 cm^−1^and 1294 cm^−1^ were attributed to symmetric stretching of C–O in monomers. A weak shoulder at 1246 cm^−1^ was assigned to N–H deformation stretching. A broad band around 930–1225 cm^−1^ showed asymmetric stretching of C–O–C and Si–O stretching vibration due to presence of silicates in the constituents. A small band obtained in the region 730–850 cm^−1^ was attributed to C–N–H asymmetric stretching in polymer matrices. In Figure 1b–d after curing different IR spectra were obtained. The low intense peak at 1711 cm^−1^ corresponded to free carbonyl group till 24 h. A similar pattern of decreasing intensity was observed for peaks around 1600 cm^−1^ and 1730 cm^−1^ and corresponded to consumption of free carbonyl during polymerization. After 24 h, a new shoulder at 1698 cm^−1^ appeared which was assigned to hydrogen bonded carbonyl C=O in composite sample. A significant decrease in intensity was observed at 1635 cm^−1^ which corresponded to C=C stretching of methacrylate group. A new shoulder appeared at 1646 cm^−1^ and a small peak at 1605 cm^−1^ formed a shoulder at 0 h. A shoulder became prominent at 1539 cm^−1^ immediately after curing and formed a small peak with time; this might be due to N–H deformation stretching. A shoulder peak at 1087 cm^−1^ formed a distinct band at 1095 cm^−1^ after curing, which was attributed to C–O–C and Si–O bonds. In the same region prominent peak, shifting and broadening was observed at 1000 cm^−1^ till 72 h (Figure 1b–f). The band at 1031 cm^−1^ became prominent after 0 h whereas a shoulder at 933 cm^−1^ became less clear with time (C–O–C and Si–O overlapping area). No considerable changes were observed after 48 h (Figure 1e) overall in the spectra.

**Figure 2:**
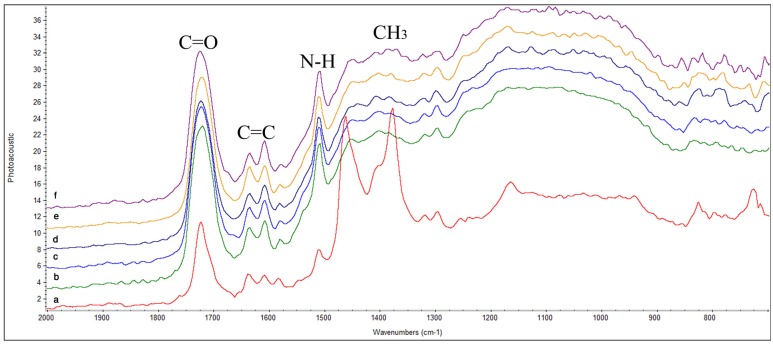
FTIR spectra of TPH showing peaks for different functional groups at different time intervals; (**a**) before curing; (**b**) after curing 0 h; (**c**) 8 h; (**d**) 24 h; (**e**) 48 h; (**f**) 72 h.

**TPH:** In Figure 2a, symmetric and asymmetric C–H stretching of the methyl groups was observed at 2890 cm^−1^ and 2930 cm^−1^. Before curing, the spectra of TPH contained a peak for free carbonyl (C=O) group at 1711 cm^−1^. At 1638 cm^−1^ C=C stretching vibration of the methacrylate group was visible, while C=C in aromatic ring of benzene present in resin matrices was seen at 1610 cm^−1^. Peak for N–H deformation stretching of UDMA appeared at 1507 cm^−1^. Similarly, peaks attributed to asymmetric and symmetric stretching of methyl group appeared at 1430–1470 cm^−1^ and 1370–1380 cm^−1^, respectively. The C–O stretch peak was prominent at 1176 cm^−1^. After curing, (Figure 2a) a profound increase in intensity of peak at 1711 cm^−1^ was observed due to conversion of free carbonyl into bonded carbonyl. Decrease in peak intensity of aliphatic C=C and increase in aromatic C=C was observed after curing at 0 h. The increase in intensity of aromatic C=C peak was due to the phenomenon of cyclization associated with polymerization. Peak for N–H showed pronounced increase in its intensity at 0 h after curing which led to appearance of a broad band instead of a sharp peak that could be attributed to methyl group at 1260–1470 cm^−1^. From 0 h to 72 h (Figure 2b–f), no further significant change was found in the spectra.

**Figure 3:**
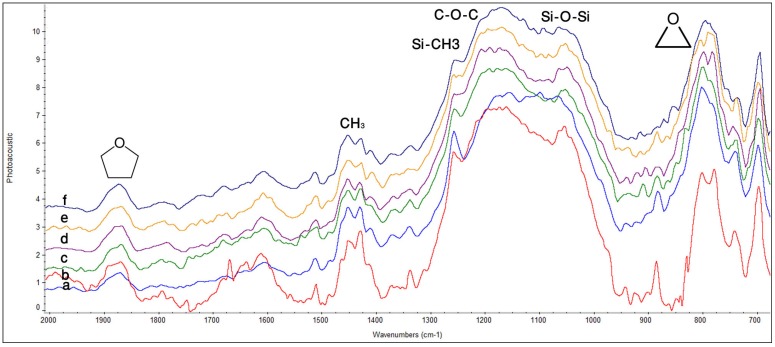
FTIR spectra of P90 showing peaks for different functional groups at different time intervals; (**a**) before curing; (**b**) after curing 0 h; (**c**) 8 h; (**d**) 24 h; (**e**) 48 h; (**f**) 72 h.

**P90:** In Figure 3a, the peak contributing to oxirane functional group was found at 800–890 cm^−1^ before photo-polymerization. Similarly, an absorption band attributed to siloxane (Si–O–Si) was found at 1055–1020 cm^−1^. Peak for Si–CH_3_ was identified as a weak hump at 1260 cm^−1^. The C–O–C stretching peak was found around 1140–1070 cm^−1^. Peak attributed to asymmetric bending of methyl group at 1430 cm^−1^ was also found. Peak for epoxy from silane coupling agent appeared at 1870 cm^−1^. Peak for methylene asymmetric stretching was observed at 2921 cm^−1^. In Figure 3b, sharpening of oxirane peak accompanied by stretching vibration was observed at 800–890 cm^−1^ after curing at 0 h. Similarly, peaks at 1099 cm^−1^ (Si–O–Si), 1155 cm^−1^ C–O–C) and 1257 cm^−1^ (Si–CH_3_) shifted, and a pronounced increase in intensity was observed. A shoulder peak attributed to methyl symmetric bending became visible after curing at 1380 cm^−1^. Inter-conversion between symmetric and asymmetric methyl peak was observed from 0 h to 72 h in Figure 3b–f. A sharp increase in peak for methylene (CH_2_) at 2919 cm^−1^ accompanied by stretching asymmetric vibration were observed. Lifting of band attributed to C–O–C and Si–CH_3_ was seen, which could be due to exhaustion of previously available Si–O–Si group due to their increased bonding with oxirane group. Molecular rearrangement was observed throughout the spectra from 0 h to 72 h due to the phenomenon of intra-molecular cross-linking between oxirane and Si–O–Si group. 

### 2.2. Atomic Force Microscopy (AFM)

**Figure 4:**
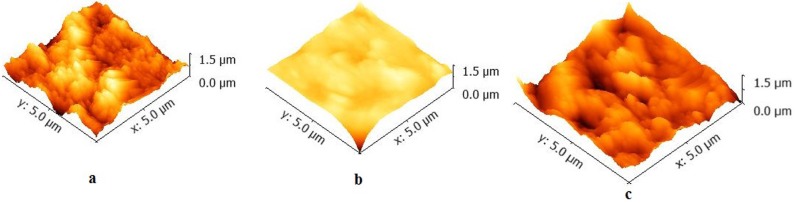
AFM images of (**a**) Z350; (**b**) TPH; (**c**) P90 showing surface morphology without bacterial adhesion.

The surface roughness was analyzed by using AFM and the three dimensional images are given in Figure 4a–c and the R_a_ and R_ms_ values, and minimum grain size is given in Table 1.

**Table 1:** Surface roughness values and grain size of commercial composite materials.

Samples	Surface Roughness (nm) R_a_/R_ms_	Minimum grain size (µm)
Z350	64.1/89.1	0.24
TPH	166/235	0.27
P90	54.4/66.4	0.32

### 2.3. Contact Angle Measurement

The obtained values for hydrophobicity are; Z350 68 (S.D. ± 4.5), TPH 82 (S.D. ± 2.5), P90 92 (S.D. ± 2.2). According to this data, P90 seems to be more hydrophobic in nature as compared to TPH and Z350.

### 2.4. Bacterial Adhesion

#### 2.4.1. *S. aureus*

**Figure 5:**
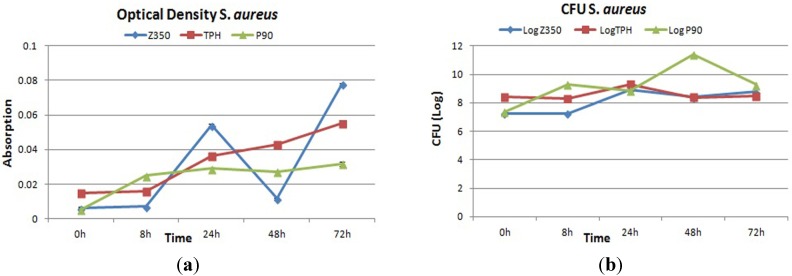
(**a**) Absorbance and (**b**) log values of colony forming unit count of Z350, TPH and P90 with *S. aureus.*

**Figure 6:**
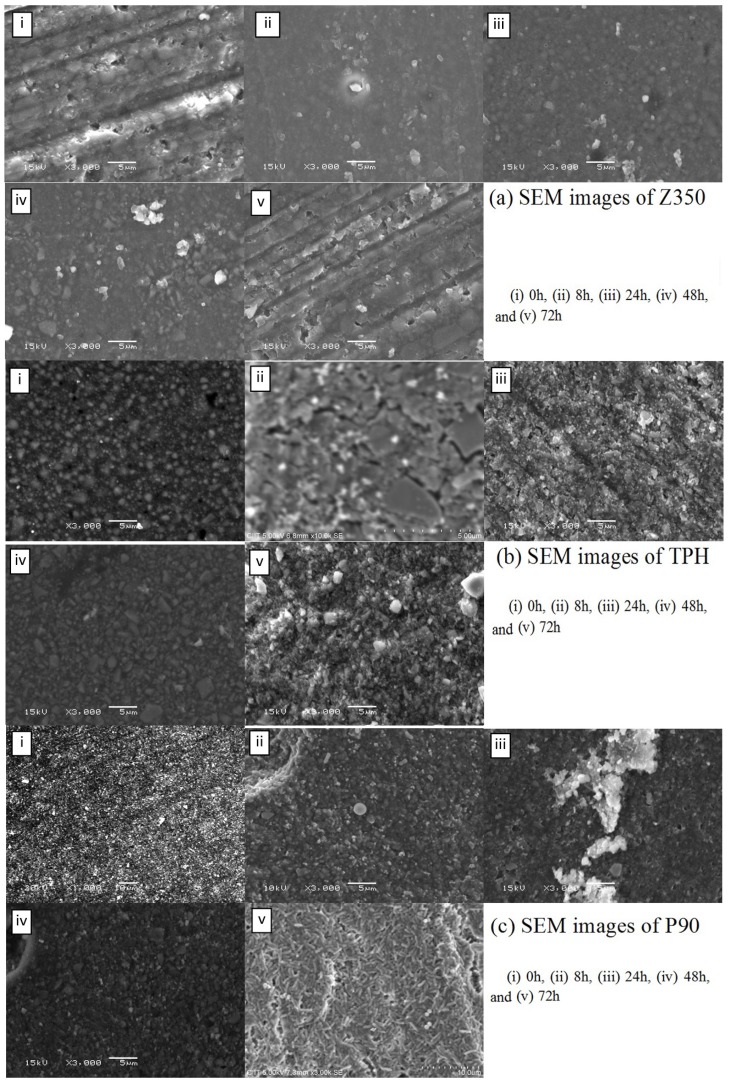
SEM images of (**a**) Z350; (**b**) TPH; and (**c**) P90 with *S. aureus.*

Figure 5a showed the optical density values of all commercial composites with *S. aureus*. Periodic surge was observed in absorbance value for bacterial colonies in case of Z350 with greater values for absorbance at 24 h and 72 h followed by a sharp decline at 48 h, whereas, for TPH, constant values of absorbance were observed from 0 to 8 h. After 8 h, a substantial increase in absorbance values was observed and values continued to rise till 72 h. In case of P90, after an initial rise in absorbance values from 0 to 8 h, the value for absorbance became almost constant from 24 to 48 h. The statistical analysis showed that there was insignificant difference among these groups. Figure 5b showed the log value of Z350, TPH and P90 with *S. aureus*. The values for Z350 exhibited that from 0 to 8 h no significant increase in the number of colonies was acquired from dislodged bacteria. At 24 h incubation, the number of colonies showed a rise. At 48 h incubation, a decline in number of colonies was seen with slight elevation in their number at 72 h incubation period. For TPH, the initial log value of CFU was more or less retained till 8 h incubation period. After 8 h, a gradual increase in number of colonies could be observed till 24 h incubation period. After 24 h, a slight trench in CFU log value was seen. This value was found to be almost constant till 72 h of incubation period. The CFU log value of P90 was constantly on the rise, starting from 0 h to 8 h incubation period. However, after 8 h, a slight decline in log value for CFU was seen, which began to rise again after 24 h, reaching a maximum value for the number of colonies at 48 h. After 48 h, a sudden plummet in log value was registered. SEM images showed no significant adhesion of *S. aureus* cells on dental composites from 0 to 72 h as shown in Figure 6a–c.

#### 2.4.2. *P. aeruginosa*

**Figure 7:**
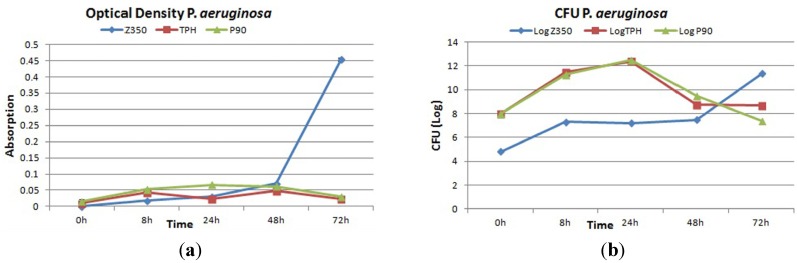
(**a**) Absorbance and (**b**) log values of colony forming unit count of Z350, TPH and P90 with *P. aeruginosa.*

**Figure 8:**
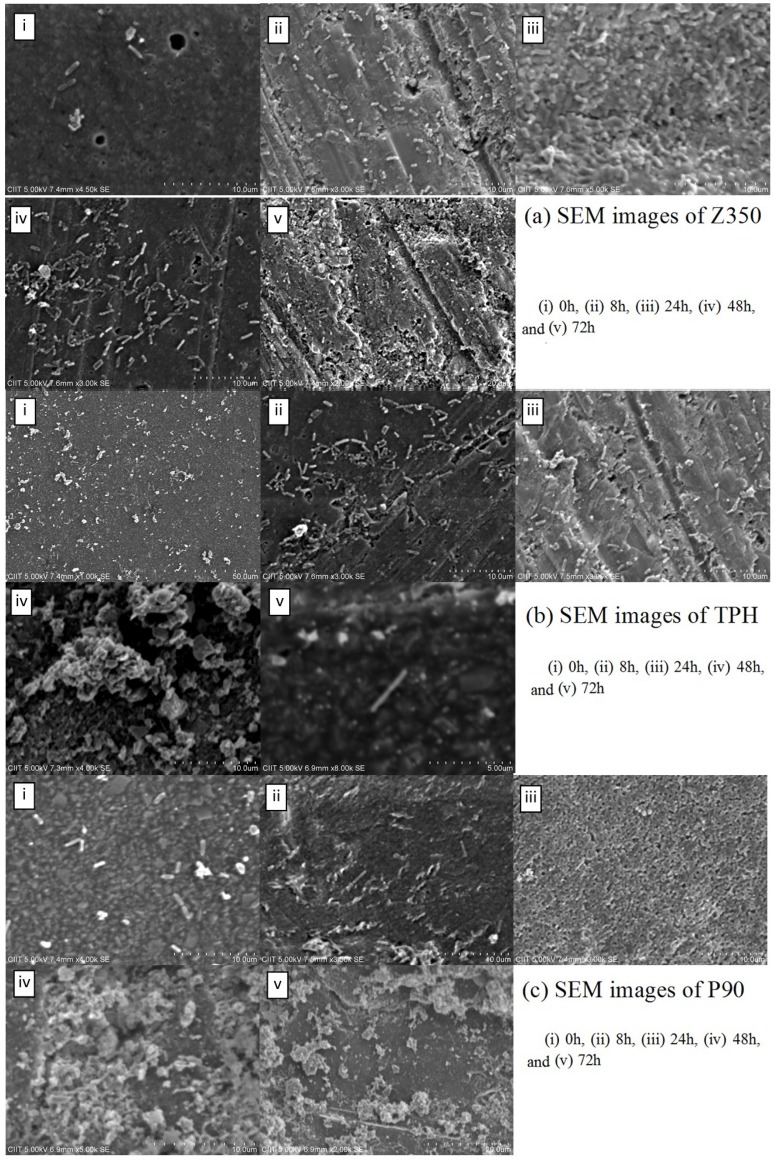
SEM images of (**a**) Z350, (**b**) TPH, and (**c**) P90 with *P. aeruginosa.*

Figure 7a showed the absorbance values of all commercial materials with *P. aeruginosa*. For Z350, a slow and gradual increase of absorbance values was observed till 48 h followed by a sharp surge which continued till 72 h. Periodic surge was observed in the absorbance value for bacterial colonies in case of TPH with greater values for absorbance at 8 h and 48 h. In case of P90, a moderate increase in absorbance value from 8 h to 24 h was registered with maximum a value at 24 h. After 24 h, a decline in absorbance value was observed till 72 h. There was no statistical difference among these groups. Figure 7b showed gradual rise in CFU log value of Z350 was seen from 0 to 8 h of incubation. A constant log value was seen from 8 h to 48 h of incubation. After 48 h, a sharp increase in log value was seen showing active proliferation in number of bacterial colonies on composite material. Whereas, with TPH, constant increase in log value for CFU was seen from 0 h to 24 h of incubation. After 24 h incubation, a gradual decline in log value was obtained showing thinning up of colony density after a 24 h incubation period. P-90 shared more or less the same log values for CFU from 0 h to 24 h. After 24 h, a sharp decline in log value was spotted till 48 h followed by constant log value till 72 h. SEM images showed a good number of bacterial cells adhering to the composite surface which seemed to be retained till 72 h of incubation (Figure 8a,b). In Figure 8c, a massive increase of bacterial adhesion was observed with a continuous rise in colony number till 8 h of incubation. At 24 h, the cell size began to diminish accompanied with reduction in colony number till 72 h.

#### 2.4.3. *E. coli*

**Figure 9:**
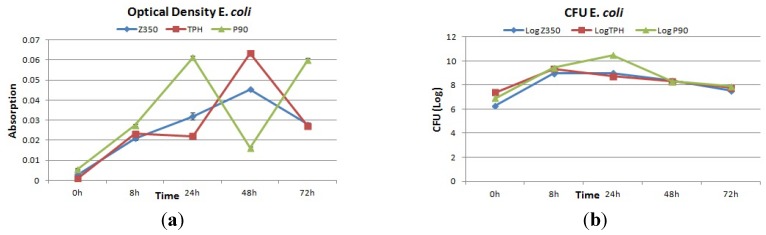
(**a**) Absorbance and (**b**) log values of colony forming units of Z350, TPH and P90 with *E. coli.*

**Figure 10:**
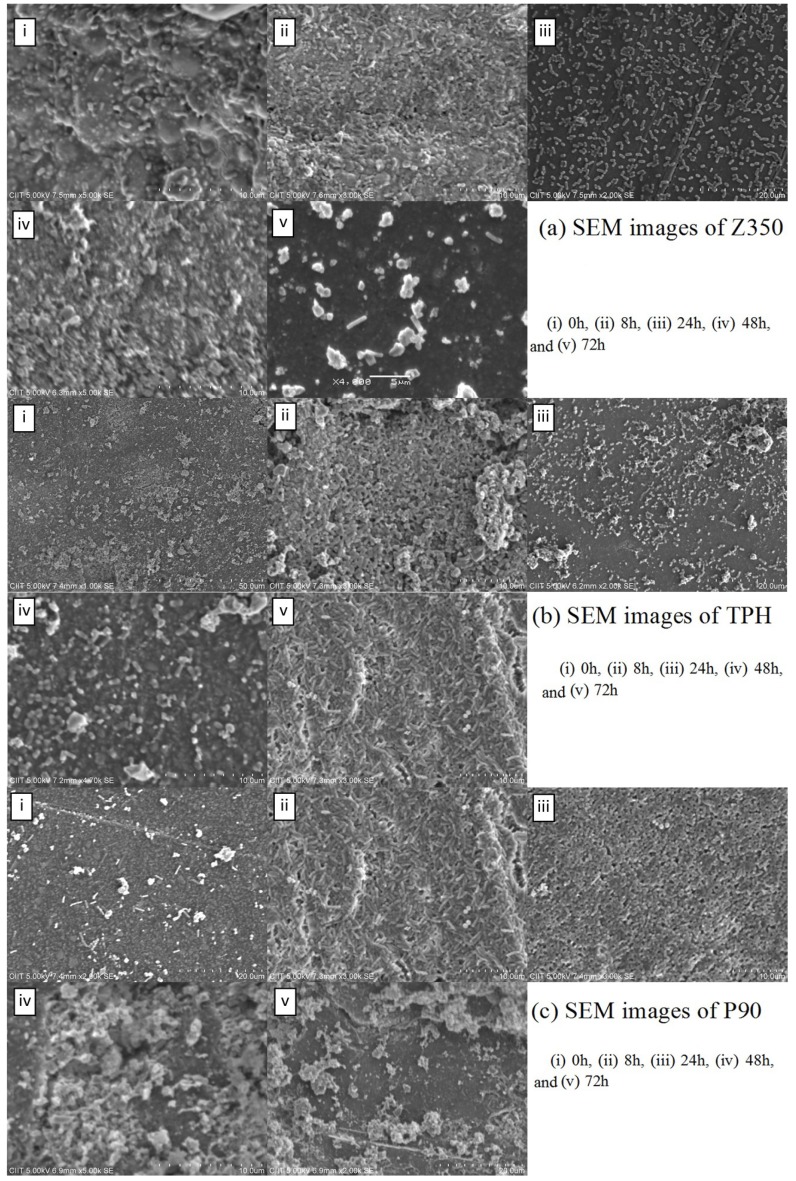
SEM images of (**a**) Z350; (**b**) TPH; and (**c**) P90 with *E. coli.*

The absorbance value of *E. coli* is shown in Figure 9a. A constant rise in absorbance values of Z350 was seen from 0 h to 48 h of incubation. A decline in absorbance values was seen at 72 h. TPH showed a constant rise with a slight dip from 0 h to 24 h in the absorbance values, followed by an increase in absorbance values at 48 h incubation. After that, again a sudden decline in absorbance values was seen at 72 h. In case of P90, the absorbance value was found to be continuously on the rise till 24 h of incubation. After 24 h, a sudden decline was registered. After 48 h of incubation, again a sharp increase in absorbance values was seen till 72 h. An insignificant difference was observed in these groups. Figure 9b shows an increase in log value was observed from 0 h to 24 h for Z350. After 24 h, log values decreased gradually till 72 h. After an initial increase of log values from 0 h to 8 h for TPH samples, they continuously declined till 72 h of incubation. P-90 showed the highest log value for CFU at 24 h adopting an almost constant rise in log value from 0 h to 8 h of incubation. After 24 h, the log value showed a trench, continuing from 48 h to 72 h. SEM images showed a large number of initial bacterial attachments followed by a rise in number of bacterial colonies till 24 h, as shown in Figure 10a. The number started to recede after 48 h showing little adhesion on the composite surface. In Figure 10b, a significant number of bacterial adhesions was found at 8 h–24 h, and then showed a decrease in colony numbers. A large number of initial bacterial attachments followed by an intensive proliferation rate till 8 h of incubation period were observed in Figure 10c. At 24 h, cell size began to diminish and the number of bacterial colonies became less.

## 3. Discussion

It is very important to consider those characteristics that inhibit surface adhesion and proliferation of bacteria. Apart from the in-depth adhesion study of oral micro-biota involved in dental disease, information on chemical and physical characterization of the dental restorative material becomes indispensible. In this study, we investigated the adhesion of three bacterial strains on newly developed commercial dental restorative materials (Z350, TPH and P90) with respect to structural analysis, surface roughness and wettability, which was not investigated before. Knowledge of the inherent characteristics of dental materials is important as these can affect the manipulation and the overall properties of the materials [25].

Degree of polymerization associated with C=C peak of methacrylates in composites, *i.e.*, TPH and Z350, showed inter-conversion between aliphatic and aromatic C=C. Increase in peak intensity for aromatic C=C, with the passage of time after curing, depicted phenomenon of cyclization [26,27,28] and led to the availability of NH group and encapsulation of CH_3_ group. This could be explained as a plausible cause for the meager number of bacterial cells adhering to these composite surfaces. The observed bacterial growth could be due to attachment site provided by NH group. After curing, CH_3_ groups were found stitched into the polymer matrix and not as many adhesion sites were available as compared to P90. On the other hand, P90 afforded the availability of CH_3_ groups due to a dynamic molecular arrangement in the silorane region [29,30]. Adhesion of bacteria is also dependent on the strain type along with availability of certain functional groups. *S. aureus* did not show much adhesion despite the availability of CH_3_ after photo-curing on P90 composite surface*. P. aeruginosa* and *E. coli* on the other hand showed massive attachment and adhesion on P90. 

From earlier studies on Contact Angle Measurement of bacterial strains such as *E. coli, S. aureus* and *P. aeruginosa,* it was found that *E. coli* have relatively higher values for hydrophobicity as compared to *S. aureus* and *P. aeruginosa* [31]. The ability of bacteria to adhere to a material surface depends on the hydrophobicity of both bacteria and material surfaces. Hydrophilic materials are more resistant to bacterial adhesion than hydrophobic materials [32]. Accordingly, P90 and TPH possess greater contact angle measurements as compared to Z350 which was in conformity with previous studies. The selected bacterial strains are endemic to oral cavity, and play a vital role in biofilm formation on tooth and biomaterial surfaces; however, similar work has been done on closely related strains such as *S. epidermidis* and *Streptococcus mutans* with other restorative materials [4,33,34].

Due to the presence of Silorane in P90, it was expected that a lower number of bacterial adhesion would be yielded but, in this study, our SEM images suggested otherwise. The number of bacterial adhesions was higher on hydrophobic material (P90) compared to relatively hydrophilic material (Z350 and TPH) over a period of time (*i.e.*, 0 h, 8 h, 24 h, 48 h, and 72 h) due to the absence of shear force. Shear force happens to be a significant feature of oral environments that aids in defining biofilm patterning. Biofilms subjected to shear force are thin and dense [32,35]. The actual number of adhesions observed with SEM was reciprocal to the Colony Forming Unit Count and Optical Density value. Based on this, an inverse relationship between the number of bacterial cells showing initial attachment/loose adhesion to the number of bacterial cells firmly adhering to the biomaterial surface can be assumed. This relationship can only be established *in vitro* due to the presence of limiting factors such as food and space. On the other hand, surface roughness does not seem to contribute to bacterial adhesion as expected. Bollen *et al.* [14] also reported the absence of a link between surface roughness and plaque formation and retention (*i.e.*, microbial adhesion). Grain size seemed to play a significant role in bacterial attachment and adhesion. Composite material (P90) having larger grain (0.32 µm) size showed greater bacterial adhesion as compared to composite material (Z350) having smaller grain size (0.24 µm) [36]. The critical period for bacterial adhesion was 0 h–8 h after curing which yielded functional groups (CH_3_ and NH) and it is expected that the bacterial adhesion depends on the material surface chemistry as well as bacterial cell surface chemistry and particle size [37]. Moderate number of bacterial adhesion was seen on TPH and the least number of bacterial adhesions was observed on Z350. In case of *P. aeruginosa*, Z350 showed minimal bacterial adhesion in comparison to the other two materials; the number of bacterial cells remained more or less constant over the time period. Optical density and CFU values, on the other hand, showed a constant increase in the number of bacterial cells with the passage of time. This might be due to the nano-nature of composite particles of Z350 and lesser value for Contact Angle Measurement. Since space was not the limiting factor for those cells that succeeded in attaching to the surface of Z350, these cells survived and continued to proliferate. P90 showed a massive number of bacterial cell adhesions, but due to limiting factors, such as space, the number of bacterial colonies showed a sharp recession. In case of TPH, a clear reciprocal relationship between the number of loosely attached bacterial colonies and those that showed firm adhesion was established. *S. aureus* showed insignificant adhesion on all three types of composite materials owing to its moderately hydrophobic nature in contrast to the relatively hydrophilic nature of Z350 and TPH. P90 however showed meager adhesion with *S. aureus* which can be attributed to its hydrophobic nature. TPH showed infinitesimal attachment whereas Z350 showed almost no attachment or adhesion with this particular strain*.* This means that the material surface of these composites (Z350 and TPH) does not provide a suitable habitat for this particular species owing to its surface charge, morphology and composition. The monomer leaching with the passage of time seems to have a pronounced adverse effect on the growth rate of *S. aureus* as compared to the other two strains. Further insight could be obtained with DNA and protein level studies as to how leaching of TEGDMA, Bis-GMA, UDMA monomers is affecting the genetic and cellular makeup of *S. aureus* only and not the other two strains [11].

These findings suggest that Z350 (nano-composite) has a better chance of evasion of biofilm formation as compared to P90 and TPH. Although oral microbiota are not restricted to these three strains that we have used in our study, they are still quite significant in the establishment of biofilm and progression of disease and infection in oral environments. Therefore, a composite material able to combat against adhesion from these strains may well be able to prevent adhesion from other bacterial strains as well.

## 4. Materials and Methods

Three commercial dental restorative materials [Filtek™ Z350 (Z350) and Filtek™ P90 (P90) (3M ESPE, Seefeld, Germany) and Spectrum^®^TPH^®^ (TPH), (Dentsply, Konstanz, Germany)] were tested and their compositions are given in Table 2.

### 4.1. Sample Preparation

In this study, 200 sample discs of size 8 mm × 2 mm were prepared according to manufacturer’s instruction using PTFE molds. The composites were polymerized by using high intensity blue light (LED, wavelength ≈ 470 nm). The samples were polished with grit paper (×4000) and washed with de-ionized water and stored in air tight test tubes after drying.

### 4.2. Characterization

#### 4.2.1. Fourier Transform Infrared Spectroscopy (FTIR)

Sample discs were assessed for degree of conversion of chemical compounds before and after curing for periodical time at 0 h, 8 h, 24 h, 48 h and 72 h, using FTIR (Thermo Nicolet 6700, Thermo Fisher Scientific, Waltham, MA, USA) coupled with Photo-Acoustic sampling cell, accumulating 256 scans at 8 cm^−1^ resolution with mid-infrared range of 400–4000 cm^−1^. Sampling cell was purged with dry helium to keep the sampling chamber free of moisture. Spectral data was obtained using OMNIC 7™ software, (Thermo Scientific, Waltham, MA, USA). 

**Table 2:** Composition of commercial dental restorative composites.

Composite Material	Manufacturer	Composition*
Filtek™ Z350	3M ESPE Filtek, Germany	Resins: Bis-GMA, UDMA, TEGDMA and Bis-EMA.Filler: zirconia, nano-silica particles59.5% vol. 82% wt
Spectrum^®^TPH^®^	Dentsply, Germany	Resins: Bis-GMA-adduct TEGDMA and Bis-EMA.Fillers: barium aluminiumborosilicate
Filtek™ P90	3 M ESPE Filtek, Germany	Resins: siloxane and oxirane polymersFiller: silanized fine quartz particles and radiopaque yttrium fluoride.55% vol. 76% wtSilane coupling agent with epoxy layer

Notes: * Bis-GMA: 2,2-Bis[4-(2-hydroxy-3-methacryloyloxypropoxy)-phenyl] propane with hexamethylene diisocyanate); UDMA: (urethane dimethacrylate); TEGDMA: (3,6-Dioxaoctamethylene-dimethacrylate); Bis-EMA: (2,2-Bis[4-(2-methacryloyloxyethoxy) phenyl]propane).

#### 4.2.2. Atomic Force Microscopy (AFM)

Surface topography of dental composites, Z350, TPH and P90 was examined by means of Molecular Imaging’s PicoPlus™ 2500, USA. Contact mode cantilevers and integrated silicon nitride tips were used. Height data from the 5 µm^2^ area images were processed using the first order flattening option and the average surface roughness was analyzed. The surface roughness (R_a_ and R_ms_) was measured. Images of the resin composites were obtained at three different locations and the average R_ms_and R_a_ roughness was determined for particular composites based on these images from different locations. The results have been included in the manuscript. The topographical images were acquired by UK Soft Software and were analyzed by Gwyddion Version 2.4. (Open Source Software covered by GNU GPL).

#### 4.2.3. Contact Angle Measurements

To evaluate the wetting properties of samples, advancing contact angle measurements of water were performed by a droplet expanding technique, using a CAM 200 Optical Contact Angle Meter (KSV Instruments Ltd., Helsinki, Finland) equipped with a video recorder that collected one image per second. Image analysis was performed with CAM 200 Software and contact angle calculation using curve fitting was based on the Young-Laplace equation, yielding contact angles on either side of the droplet and their mean value. Six samples of each composite material were cast and their contact angles were evaluated to estimate the standard deviation.

#### 4.2.4. Bacterial Strains and Growth Conditions

The Anaerobic strains, *Staphylococcus aureus* (*S. aureus* ATCC 25923), *Pseudomonas aeruginosa* (*P. aeruginosa* ATCC 27853) and *Escherichia coli* (*E. coli* ATCC 25922) were revived independently from their respective Glycerol stock cultures and sub-cultured onto LAB, UK Nutrient Agar. Incubation was done for 24 h at 37 °C in VWR Incubator and later colonies were picked and sub-cultured into LAB, UK Trypton Soy Broth (TSB) again for 24 h at 37 °C.

#### 4.2.5. Adhesion Testing

Total number of samples used for adhesion testing was 90. All tests were done in triplicate. Twelve welled Tissue Culture Test-plates were used to test each sample disc. In each well, 2 mL of TSB along with test material disc and 20 µL of bacterial culture was placed. Each sample disc was tested with all three types of selected bacterial strains so there were three different types of bacterial culture which were used for inoculation of the test materials. Culture plates were placed in VWR incubator, USA at 37 °C and periodic characterizations of test materials were done at 0, 8, 24, 48 and 72 h.

After the adhesion experiments, each sample disc was vortexed in Bio-Rad BR-2000 vortex, USA for 3 min at 3000 rpm in a test tube containing 0.9% Normal saline to dislodge adherent cells. Tenfold serial dilution of the vortexed solutions were inoculated on Nutrient Agar plates, and the number of adherent bacterial colonies (colony forming unit (CFU) was counted after 24 h of incubation at 37 °C). Optical Density of that saline solution with bacterial cells was measured in Thermo Spectronics GeneSys (Thermo Scientific, Waltham, MA, USA), 10 UV-Spectrophotometer at 600 nm. To observe the significant difference between different groups, the least significant difference (LSD) test is used. The LSD test makes pair wise comparison between the different groups on the basis of well known Student’s *t* test by using pooled standard deviation of pair wise groups.

#### 4.2.6. Scanning Electron Microscopy (SEM)

After the adhesion experiment test material disc was gently rinsed with Phosphate Buffer Saline (PBS) to remove non-adherent or loosely adherent bacteria and then fixed with 2.5% glutaraldehyde (Merck, Darmstadt, Germany) for 24 h. After fixation, test samples were processed by a method of sequential dehydration. Concentrations of ethanol used for sequential dehydration were; 50%, 60%, 70%, 80%, 90% and 100% vol/vol. Incubation time was kept to 30 min for each ethanol-water solution. After sputter coating with carbon, the samples were investigated by Scanning Electron Microscopy (SEM; Hitachi Scanning Electron Microscope SU-1500, Niigata, Japan) Voltage range was from 5 to 20 kV and magnification range went up to 10,000×.

## 5. Conclusions

The grain size of composite materials seems to be directly proportional to the number of bacterial cell adhesions. This means the larger the grain size, the greater the propensity of bacterial attachment and adhesion. Similarly, Contact Angle Measurement also provided us with an indication that specific surface types are susceptible to adhesion and attachment by a specific range of bacterial strains. The degree of polymerization after curing at specific time intervals led to identification of specific functional groups that aid in bacterial adhesion.

## References

[1] Khan A.S., Tahir M.T., Khan M., Mian S.A., Rehman I.U. (2015). An update on glass fiber dental restorative composites: A systematic review. Mater. Sci. Eng. C.

[2] Grossman E., Matejka J. (1995). Amalgam restoration and *in vitro* caries formation. J. Prosthet. Dent..

[3] Mejare B., Mejare I., Edwardsson S. (1979). Bacteria beneath composite restorations—A culturing and histobacteriological study. Acta. Odontol. Scand..

[4] Mjor I. (1997). The reasons for replacement and the age of failed restorations in general dental practice. Acta. Odontol. Scand..

[5] Merrett M., Elderton R. (1984). An *in vitro* study of restorative dental treatment decisions and dental caries. Br. Dent. J..

[6] Mjor I., Moorhead J., Dahl J. (2000). Reasons for replacement of restorations in permanent teeth in general dental practice. Int. Dent. J..

[7] Mjor I., Toffenetti F. (1992). Placement and replacement of resin-based composite restorations in Italy. Oper. Dent..

[8] Mo S-S., Bao W., Lai G-Y., Wang J., Li M-Y. (2010). The Microfloral Analysis of Secondary Caries Biofilm around Class I and Class II Composite and Amalgam Fillings. BMC Infec. Dis..

[9] Lovegrove J.M. (2004). Dental plaque revisited: Bacteria associated with periodontal disease. J. N. Z. Soc. Periodontol..

[10] Mack D., Becker P., Chaterjee I., Dobinsky S., Knoblock J.K.M., Peters G., Rohde H., Hermann M. (2004). Mechanisms of biofilm formation in Staphylococcus epidermidis and Staphylococcus aureus: Functional molecules, regulatory circuits, and adaptive responses. Int. J. Med. Microb..

[11] Orstavik D., Orstavik J. (1976). *In vitro* attachment of Streptococcus sanguis to dental crown and bridge cements. J. Oral. Rehabil..

[12] Hansel C., Leyhausen G., Mai U.E.H., Geurtsen W. (1998). Effects of various resin composite (co)monomers and extracts on two caries-associated micro-organisms *in vitro*. J. Dent. Res..

[13] Fang L., Jihua C., Zhiguo C., Ling Z., Yuhong X., Ming F., Sai M. (2009). Effects of a dental adhesive incorporating antibacterial monomer on the growth, adherence and membrane integrity of *Streptococcus mutans*. J. Dent..

[14] Bollen C.M.L., Lambrechts P., Quirynen M. (1997). Comparison of surface roughness of oral hard materials to the threshold surface roughness for bacterial plaque retention: A review of the literature. Dent. Mater..

[15] Beighton D. (2005). The complex oral microflora of high-risk individuals and groups and its role in the caries process. Commun. Dent. Oral Epidemiol..

[16] Oluremi B.B., Osungunna M.O., Idowu O.A., Adebolu O.O. (2010). Evaluation of anticaries activity of selected mouthwash marketed in Nigeria. Trop. J. Pharm. Res..

[17] Scheie A.A. (1994). Mechanisms of Dental Plaque Formation. Adv. Dent. Res..

[18] Castro P., Tovar J.A., Jaramillo L. (2006). Adhesion of *Streptococcus mutans* to salivary proteins in caries-free and caries-susceptible individuals. Acta. Odontol. Latinoam..

[19] Ilie N., Hickel R. (2011). Resin composite restorative materials. Aus. Dent. J..

[20] Harris L.G., Foster S.J., Richard R.G. (2002). An introduction to *Staphylococcus aureus*, and techniques for identifying and quantifying *S. aureus* adhesions in relation to adhesion to biomaterials: Review. Eur. Cell. Mater..

[21] Hassan-Olajokun R.E., Folarin A.A., Olaniran O., Umo A.N. (2008). The prevalent bacterial isolates of dental caries in school age children attending the dental clinic of oauthc, ile-ife. Afr. J. Cln. Exper. Microbiol..

[22] Rothbaum R., McAdams A.J., Giannella R., Partin J.C. (1982). A clinicopathologic study of enterocyte-adherent Escherichia coli: A cause of protracted diarrhea in infants. Gastroenterology.

[23] Loesche W., Syed S. (1973). The predominant cultivable flora of carious plaque and carious dentine. Caries Res..

[24] Marshall S.J., Marshall G.W. (1992). Dental amalgam: The materials. Adv. Dent. Res..

[25] Drucker D., Lilley J., Tucker D., Gibbs A. (1992). The endodontic microflora revisited. Microbios.

[26] Imazato S., McCabe J.F., Tarumi H., Ehara A., Ebisu S. (2001). Degree of conversion of composites measured by DTA and FTIR. Dent. Mater..

[27] Younas B., Khan A.S., Muzaffar D., Hussain I., Chaudhry A.A., Rehman I.U. (2013). *In situ* reaction kinetic analysis of dental restorative materials. Eur. Phys. J. Appl. Phys..

[28] Meenakshi K., Sudhan E., Kumar S., Umpapathy M. (2011). Development of siloxane based tetraglycidyl epoxy nanocomposites for high performance applications—Study of the mechanical, thermal, water absorption and flame retardant behaviour. Silicon.

[29] Lee M.H., Brass D., Morris R., Composto R.J., Ducheyne P. (2005). The effect of non-specific interactions on cellular adhesion using model surfaces. Biomaterials.

[30] Hamadi F., Latrache H. (2008). Comparison of contact angle measurement and microbial adhesion to solvents for assaying electron donor and electron acceptor (acid-base) properties of bacterial surface. Coll. Surf. B Biointer..

[31] An Y.H., Friedman R.J. (1998). Concise review of mechanisms of bacterial adhesion to biomaterial surfaces. J. Biomed. Mater. Res..

[32] Matasa C. (1995). Microbial attack of orthodontic adhesives. Am. J. Orthod. Dentofacial. Orthop..

[33] Kidd E.A.M., Beighton D. (1996). Prediction of Secondary Caries around Tooth-colored Restorations: A Clinical and Microbiological Study. J. Dent. Res..

[34] Katsikogianni M.G., Missirlis Y.F. (2010). Interactions of bacteria with specific biomaterial surface chemistries under flow conditions. Acta. Biomater..

[35] Truong V.K., Lapovok R., Estrin Y.S., Rundell S., Wang J.Y., Fluke C.J., Crawford R.J., Ivanova E.P. (2010). The influence of nano-scale surface roughness on bacterial adhesion to ultrafine-grained titanium. Biomaterials.

[36] Van Dijk J., Herkstroter F., Busscher H., Weerkamp A., Jansen H., Arends J. (1987). Surface-Free Energy and Bacterial Adhesion: An *in vivo* study in beagle dogs. J. Clin. Periodontol..

[37] Katsikogianni M., Missirlis Y.F. (2004). Concise review of mechanisms of bacterial adhesion to biomaterials and of techniques used in estimating bacteriamaterial interactions. Eurp. Cells Mater..

